# Bibliometric analysis of clinical treatment in Hirschsprung disease: trends and emerging hotspots

**DOI:** 10.3389/fped.2026.1964056

**Published:** 2026-09-22

**Authors:** Yong Liu, Rui Li, Daqing Sun

**Affiliations:** Department of Pediatric Surgery, Tianjin Medical University General Hospital, Tianjin, China

**Keywords:** bibliometric analysis, clinical treatment, hirschsprung disease, regenerative medicine, surgical techniques

## Abstract

**Background:**

Hirschsprung disease (HSCR) is a congenital gastrointestinal malformation defined by the absence of enteric ganglion cells in the distal bowel. Although surgical resection remains the primary treatment, the field has witnessed rapid evolution toward precision minimally invasive strategies, including laparoscopic/robotic-assisted procedures and standardized perioperative protocols. The expanding literature volume necessitates a systematic synthesis of the research landscape.

**Methods:**

We retrieved HSCR clinical treatment publications from Web of Science Core Collection and Scopus (2005–2025). After deduplication, 3,816 publications (3,201 articles, 615 reviews) were analyzed using CiteSpace 6.4.R1 and VOSviewer 1.6.20, covering annual trends, collaboration networks, journal distribution, reference bursts, and keyword bursts.

**Results:**

Annual output rose from 103 (2005) to 313 (2025), exceeding 200 since 2019. The United States (27.2%), China (13.0%), and the United Kingdom (8.2%) led contributions. Nationwide Children's Hospital and Huazhong University of Science and Technology were top institutional producers, while Journal of Pediatric Surgery was the most productive journal. Reference burst analysis identified ERNICA rectosigmoid HSCR guidelines (strength = 25.65) and HAEC management guidelines (strength = 23.55) as landmark works. Keyword bursts revealed three evolutionary phases: surgical technique optimization (2005–2012, dominated by “surgical technique,” strength = 18.96); pathophysiological and experimental therapies (2011–2018, with “stem cell” and “transplantation”); and guideline-driven standardized management (2019–present, with active bursts in “guidelines,” strength = 8.53; “colorectal surgery,” strength = 10.68; and “prematurity,” strength = 7.89). Persistent bursts in colorectal surgery and guideline-related terms indicate sustained attention, while stem cell-based regenerative therapies remain at an early experimental stage and represent an emerging frontier, although clinical translation has not yet been demonstrated.

**Conclusions:**

HSCR clinical treatment research has grown substantially over two decades, transitioning from surgical technique optimization through experimental therapies to standardized multidisciplinary management. Current hotspots include HAEC prevention, long-term functional follow-up, and clinical guideline implementation—all core components of precision minimally invasive frameworks. Stem cell-based regenerative therapies (still at a preclinical stage) and precision surgery for long-segment HSCR are future directions warranting further investigation. These findings provide an evidence-based reference for navigating the evolving landscape and prioritizing investigations.

## 1 Introduction

Hirschsprung disease (HSCR) is a congenital gastrointestinal malformation characterized by abnormal development of the enteric nervous system, with the core pathological feature being the complete absence of ganglion cells in the submucosal and myenteric plexuses of the distal intestinal wall. This congenital disorder, resulting from failed neural crest cell migration, renders the affected bowel segment incapable of normal peristalsis, leading to functional intestinal obstruction (1). HSCR is the most common congenital dysmotility disorder of the gastrointestinal tract, with a global incidence of approximately 1 in 5,000 live births.

The disease exhibits significant differences in sex and the extent of aganglionosis—male predominance is approximately four times that of females, although this sex difference disappears when the entire colon is involved (2). Based on the length of the aganglionic segment, HSCR can be classified into short-segment, rectosigmoid, long-segment, total colonic, and total intestinal aganglionosis.

Most children with HSCR present during the neonatal period, with typical manifestations including delayed passage of meconium (94%–98% of infants with HSCR fail to pass meconium within 24 h of birth), intractable constipation, and progressive abdominal distension. Without timely intervention, the condition may lead to severe complications such as enterocolitis, intestinal perforation, and sepsis, which can be life-threatening (3).

Currently, surgical resection of the aganglionic bowel segment remains the primary treatment modality for Hirschsprung disease, with the core objective being the removal of the aganglionic segment and restoration of intestinal continuity (4). Classic surgical procedures include the Swenson procedure (resection of the entire aganglionic segment followed by end-to-end anastomosis), the Soave procedure (endorectal mucosal dissection with a retained muscular cuff and pull-through anastomosis), and the Duhamel procedure (preservation of the anterior rectal wall with a retrorectal pull-through of the colon) (5).

With the introduction of minimally invasive techniques, laparoscopic-assisted and robot-assisted procedures have been widely applied in the treatment of HSCR, substantially enhancing surgical safety and precision (6). In 2024, separate international and Chinese guidelines and expert consensus statements on laparoscopic-assisted transanal endorectal pull-through were published.

Nevertheless, despite continuous improvements in surgical techniques, postoperative complications remain a significant clinical challenge—Hirschsprung-associated enterocolitis (HAEC), soiling, constipation, and anastomotic leakage continue to occur at considerable rates, severely affecting the growth, development, and quality of life of children with HSCR (7–9). Moreover, total colonic and total intestinal aganglionosis carry a poor prognosis due to the extensive nature of the disease and the associated therapeutic difficulties. In recent years, emerging strategies such as stem cell transplantation and enteric neural stem cell regenerative therapy have demonstrated promising application prospects, opening new avenues for the treatment of HSCR (10, 11).

Despite substantial advances in the clinical treatment of Hirschsprung disease, the volume of related research literature is vast and widely distributed, and a systematic quantitative analysis of research hotspots and developmental trends is still lacking. Bibliometrics, a quantitative analytical approach grounded in mathematics and statistics, can objectively delineate the current state of research, evolutionary trajectories, and emerging frontiers in a given field. In this study, we employed bibliometric methods to systematically review and visually analyze the literature pertaining to the clinical treatment of Hirschsprung disease, aiming to identify research hotspots, reveal developmental trends, and predict future directions, thereby providing an evidence-based reference for clinical decision-making and research prioritization.

## 2 Materials and methods

### 2.1 Study design and ethical considerations

This study was a retrospective bibliometric analysis that focused exclusively on published academic literature and did not involve any human participants, animal experiments, or clinical trials. Therefore, in accordance with relevant ethical guidelines, institutional review board approval was not required for this study.

### 2.2 Data collection

We conducted a literature search in the Web of Science Core Collection (WOSCC) and Scopus databases using a Topic Search (TS) strategy. The search was restricted to original research articles and reviews published in English. The following search terms were applied:

For WOSCC: TS = (“Hirschsprung disease” OR “Hirschsprung's disease” OR HSCR OR “congenital megacolon” OR “congenital aganglionosis” OR aganglionosis) AND TS = (treatment OR therapy OR management OR surgery OR surgical OR operation OR “pull-through” OR Swenson OR Soave OR Duhamel OR TERPT OR TEPT OR “minimally invasive” OR laparoscopic OR robotic OR colostomy OR ileostomy OR “stem cell” OR “bowel irrigation” OR enterocolitis).

For Scopus: TITLE-ABS-KEY(“Hirschsprung disease” OR “Hirschsprung's disease” OR HSCR) AND TITLE-ABS-KEY(treatment OR therapy OR management OR surgery OR surgical OR operation OR “pull-through” OR Swenson OR Soave OR Duhamel OR TERPT OR TEPT OR “minimally invasive” OR laparoscopic OR robotic OR colostomy OR ileostomy OR “stem cell” OR “bowel irrigation” OR enterocolitis).

The search timeframe was set from 2005 to 2025, and the final search was performed on July 20, 2026. All retrieved records were exported—in plain text format for WOSCC and CSV format for Scopus—including full bibliographic details and cited references for subsequent analyses.

### 2.3 Inclusion and exclusion criteria

The inclusion criteria were: (1) publicly available original research or review articles focusing on the clinical treatment of Hirschsprung disease; (2) publication language restricted to English; (3) publication year within the predefined search timeframe (2005–2025).

The exclusion criteria were: (1) studies not specifically addressing the clinical treatment of Hirschsprung disease; (2) conference abstracts, editorials, news reports, letters, and other non-research documents; (3) incomplete records; (4) duplicate publications; and (5) non-English publications.

Records were filtered at the database level by document type (original article/review), language (English), and time frame (2005–2025), and then deduplicated by DOI and title using Python.

### 2.4 Analytical methods

The plain text records exported from the Web of Science Core Collection (including full bibliographic information and cited references) and the CSV records exported from Scopus were merged, deduplicated, and cleaned using Python, yielding a standardized dataset for subsequent analyses. VOSviewer (version 1.6.20) was employed to construct collaboration networks of countries, institutions, authors, and journals. Node size reflects publication volume, while edge thickness indicates the strength of collaborative relationships. CiteSpace 6.4.R1 was used for reference burst analysis and keyword burst analysis. Its burst detection algorithm identified highly influential publications and pivotal turning-point papers through reference bursts, and detected rapidly emerging research frontiers through keyword bursts. The time-slicing parameters were set from January 2005 to December 2025, with each year treated as a single slice, while other parameters remained at default settings.

## 3 Results

### 3.1 Quantitative analysis of publication

A total of 10,327 publications were initially identified (3,341 from WOSCC and 6,986 from Scopus). After restricting records to original articles and reviews published in English between 2005 and 2025, 5,715 records remained (2,228 from WOSCC and 3,487 from Scopus). After removing 1,899 duplicate records and excluding early-access articles scheduled for 2026, 3,816 records (3,201 original articles and 615 reviews) were included in the analysis. The selection process is shown in Figure 1.

**Figure 1:**
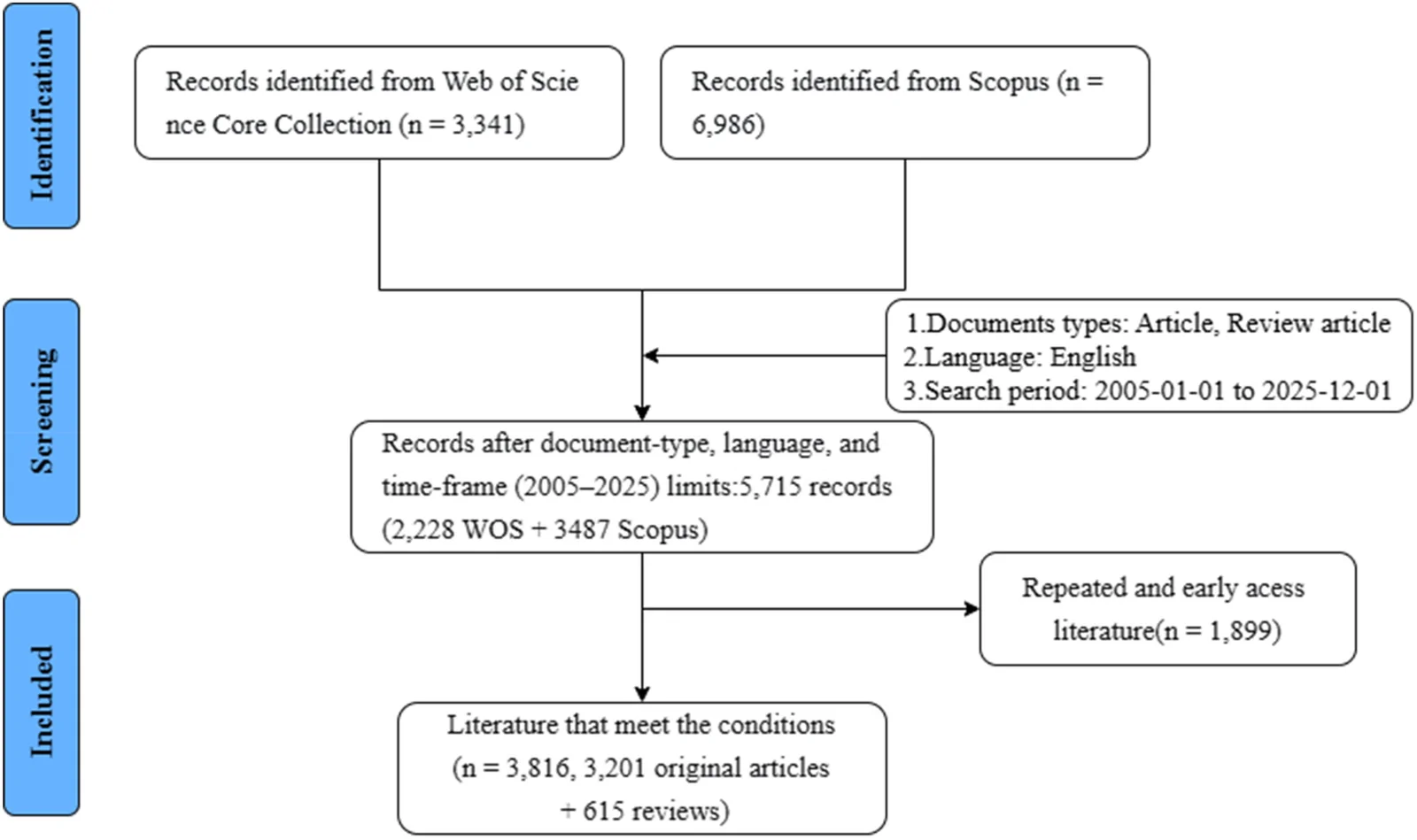
Flowchart of the literature selection process for the bibliometric analysis of Hirschsprung disease (HSCR) clinical treatment, 2005–2025. Records were retrieved from the Web of Science Core Collection (*n* = 3,341) and Scopus (*n* = 6,986). After restriction to English-language original articles and reviews (*n* = 5,715; 2,228 from WOSCC and 3,487 from Scopus), removal of 1,899 duplicate records by DOI and title, and exclusion of early-access articles scheduled for 2026, 3,816 publications (3,201 original articles and 615 reviews) were retained for analysis.

Figure 2 illustrates the annual and cumulative publication output in the field of Hirschsprung disease clinical treatment over the past two decades. The number of publications exhibited a sustained upward trajectory, increasing from 103 in 2005 to 313 in 2025, representing a nearly threefold growth. Of particular note, annual publication volumes have consistently exceeded the 200-mark since 2019, signaling robust and continuously growing scholarly interest in this domain. The year 2025 recorded the highest output, with 313 publications.

**Figure 2:**
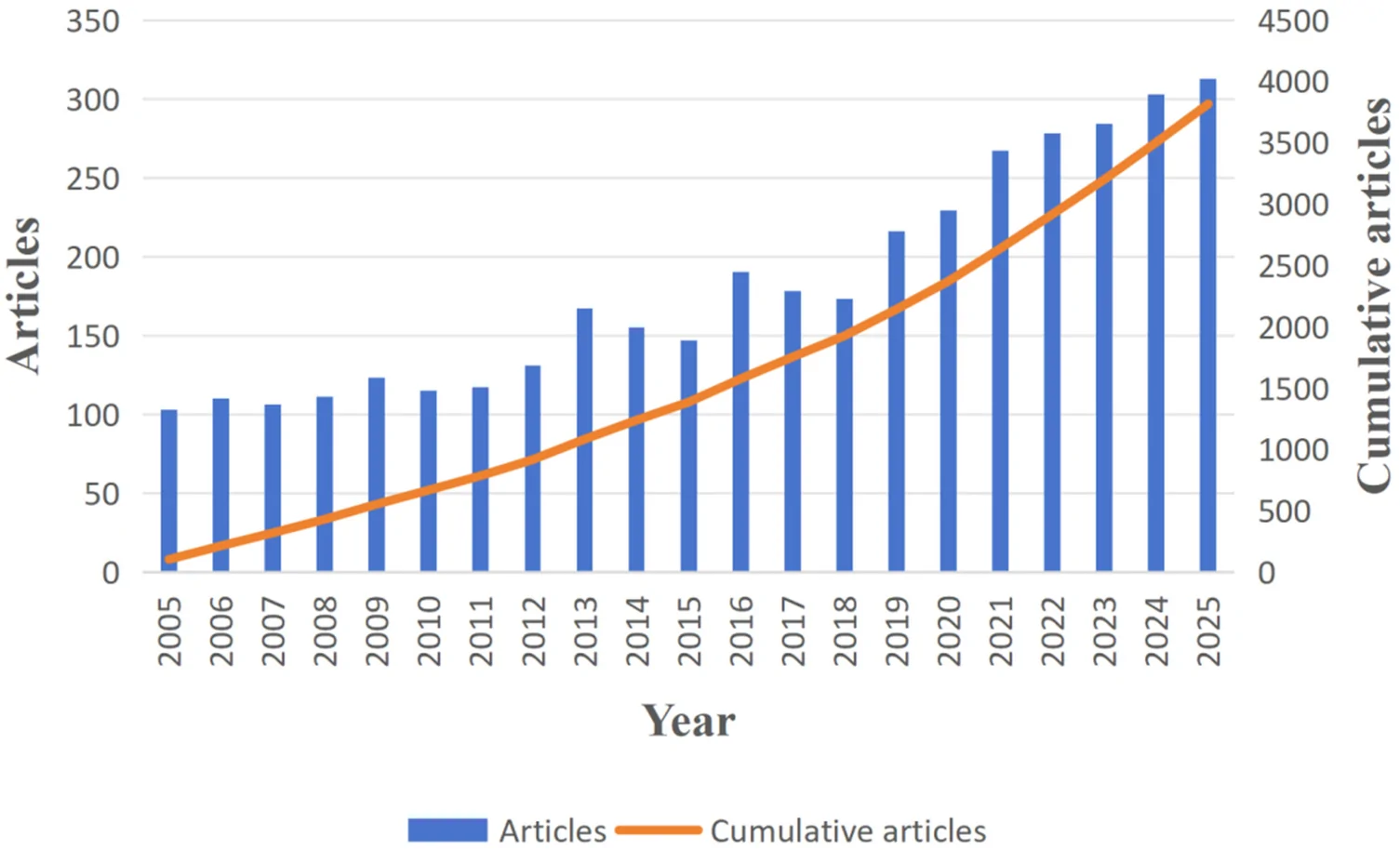
Annual and cumulative number of publications on the clinical treatment of Hirschsprung disease, 2005–2025 (*n* = 3,816). Bars (left *y*-axis) show the number of publications per year; the line (right *y*-axis) shows the cumulative number of publications. Output rose from 103 publications in 2005 to 313 in 2025, exceeding 200 per year continuously since 2019.

### 3.2 Analysis of national publication

Table 1 lists the top 10 countries by publication volume, with 7 of them located in Europe and the Americas. Publications from the top three countries account for nearly 50% of the total (*n* = 1,847, 48.4%), including the United States in North America (*n* = 1,038, 27.2%), China in Asia (*n* = 495, 13.0%), and the United Kingdom in Europe (*n* = 314, 8.2%). Using VOSviewer to filter and visualize countries with 10 or more publications, Figure 3 presents each country's collaborative network based on publication volume and relationships.

**Table 1:** Top 10 countries by number of publications on the clinical treatment of Hirschsprung disease, 2005–2025 (*n* = 3,816). “Article” indicates the number of included publications; “Citations” the total number of citations received; and “Total link strength” the cumulative strength of a country's co-authorship links with all other countries (VOSviewer 1.6.20). Countries are ranked by number of publications; percentages cited in the text are calculated against the 3,816 included records.

Rank	Country	Article	Citations	Total link strength
1	United States	1,038	28,277	390
2	China	495	6,289	111
3	United Kingdom	314	9,508	318
4	Japan	249	3,043	45
5	Italy	197	6,729	241
6	India	178	1,540	26
7	Germany	166	4,531	247
8	Canada	148	4,575	121
9	Netherlands	148	4,357	236
10	France	120	4,980	166

**Figure 3:**
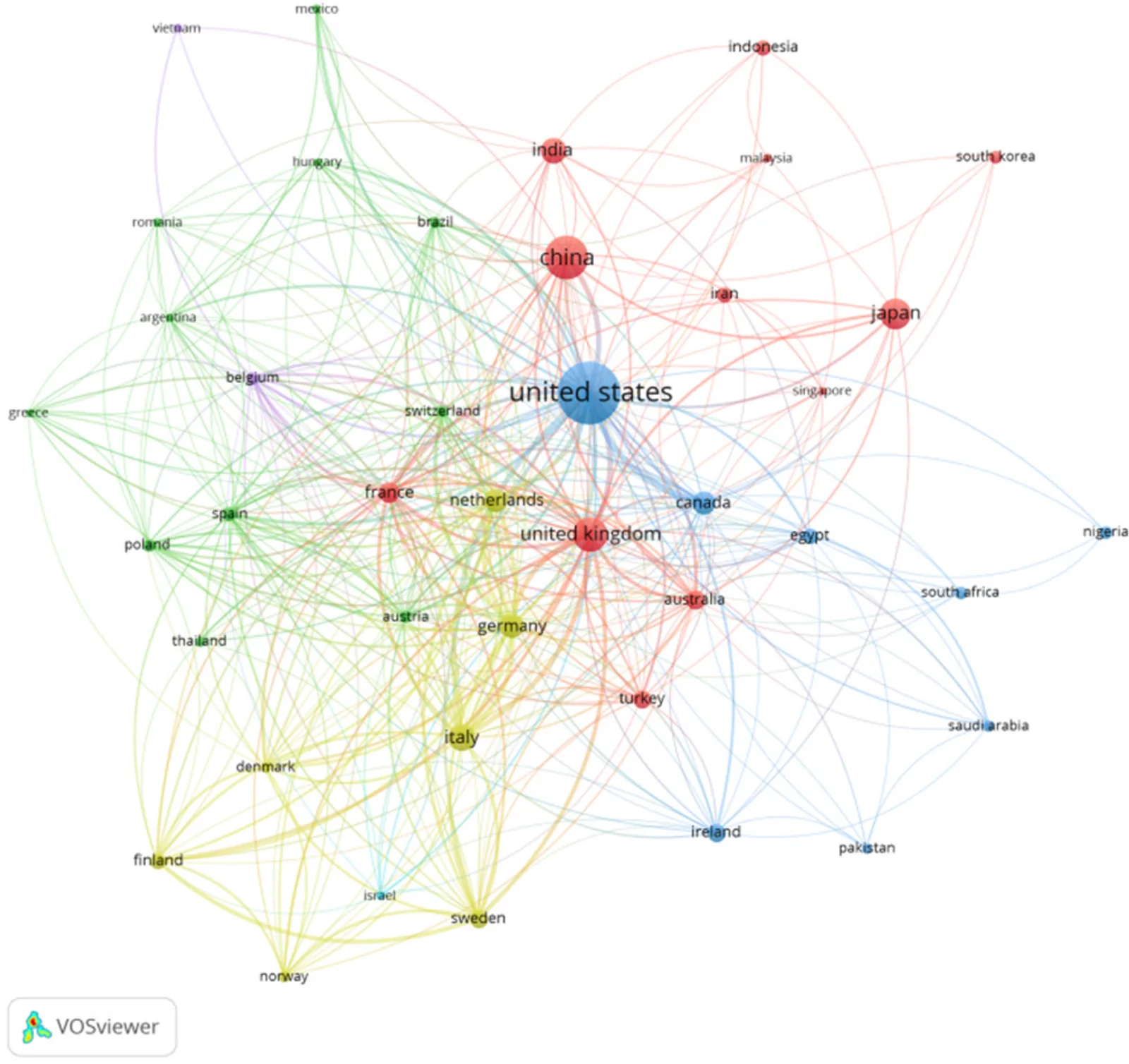
Country collaboration network for research on the clinical treatment of Hirschsprung disease, 2005–2025. The network was generated with VOSviewer 1.6.20 and includes only countries with at least 10 publications. Node size is proportional to the number of publications, node colour indicates the cluster to which the country belongs (VOSviewer clustering), and line thickness represents the strength of collaboration between countries.

### 3.3 Analysis of institution publications

Table 2 lists the top 10 institutions by publication output. The Nationwide Children's Hospital in the US published the highest number of papers at 86, followed by the Huazhong University of Science & Technology in China with 63 publications. Figure 4 displays major institutions with over 20 publications and their collaborative relationships within this field.

**Table 2:** Top 10 institutions by number of publications on the clinical treatment of Hirschsprung disease, 2005–2025. “Organization” is the institution name as indexed in the merged Web of Science Core Collection and Scopus records; “Article” indicates the number of included publications; “Citations” the total citations received; and “Total link strength” the cumulative co-authorship link strength with other institutions (VOSviewer 1.6.20). Ranked by number of publications.

Rank	Organization	Article	Citations	Total link strength
1	Nationwide Children's Hospital	86	2,150	111
2	Huazhong University of Science and Technology	63	852	4
3	University of Helsinki	48	1,913	41
4	Our Lady's Children's Hospital	46	1,167	27
5	University of Washington	46	1,449	88
6	University of Melbourne	45	1,984	34
7	University of Toronto	43	1,606	56
8	University of Utah	40	838	76
9	Harvard Medical School	38	1,115	51
10	Seattle Children's Hospital	38	1,575	72

**Figure 4:**
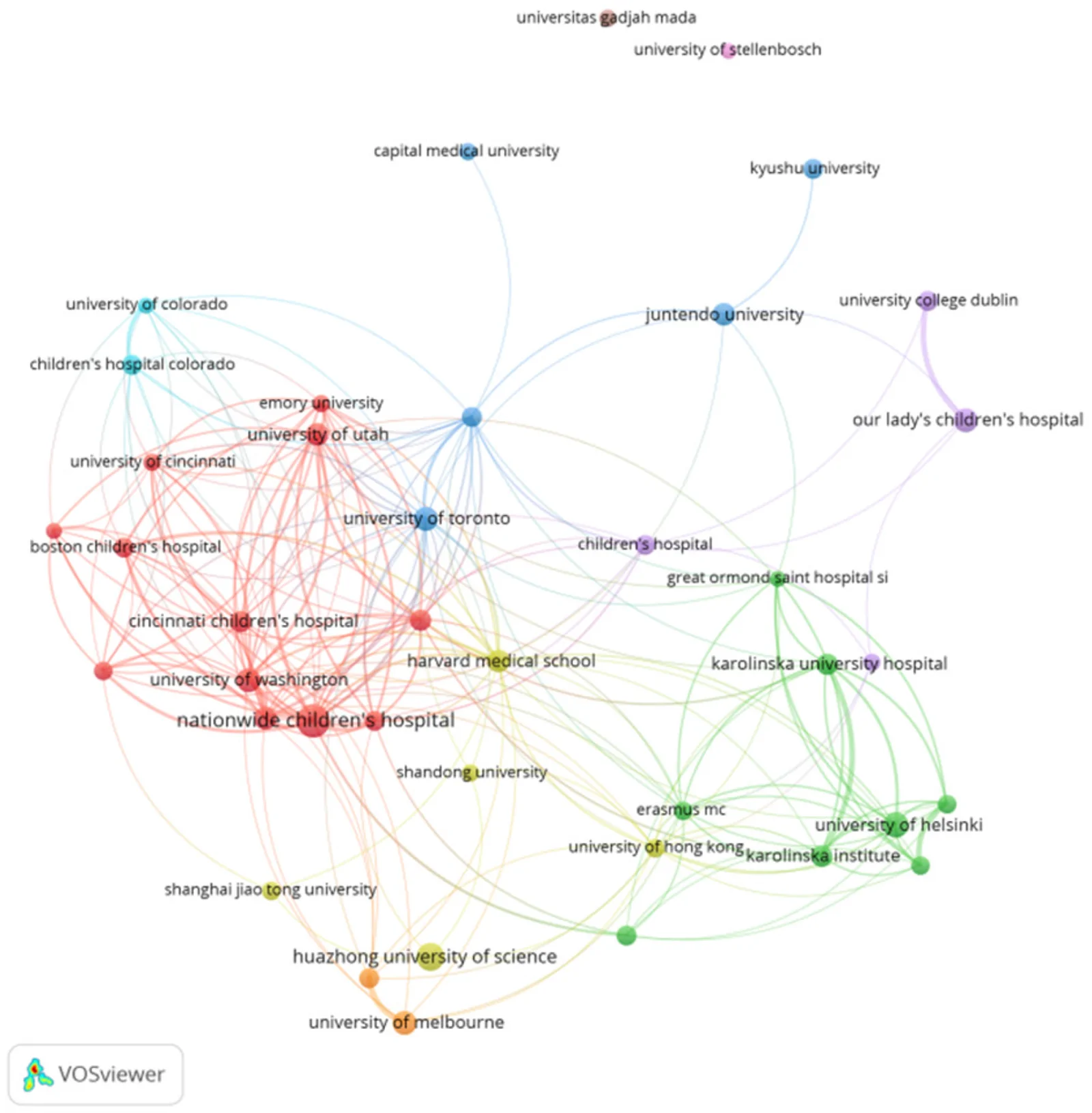
Institutional collaboration network for research on the clinical treatment of Hirschsprung disease, 2005–2025. Institutions with more than 20 publications were included. The network was generated with VOSviewer 1.6.20; node size is proportional to publication volume, node colour indicates the cluster to which the institution belongs (VOSviewer clustering), and line thickness represents the strength of collaboration between institutions.

### 3.4 Analysis of journals

Table 3 displays the top 10 journals ranked by publication volume. Journal of Pediatric Surgery leads with 454 articles and 9,160 citations, underscoring its predominant position in the field. Pediatric Surgery International ranks second with 322 articles and 5,132 citations. European Journal of Pediatric Surgery ranks third with 99 articles and 1,485 citations.

**Table 3:** Top 10 journals by number of publications on the clinical treatment of Hirschsprung disease, 2005–2025. “Article” indicates the number of included publications; “Citations” the total citations received; “IF (2025)” the 2025 Journal Impact Factor from Journal Citation Reports; and “JCR Quartile” the corresponding category quartile. Impact factors and quartiles were current as of July 22, 2026. Ranked by number of publications.

Rank	Journal	Article	Citations	IF (2025)	JCR Quartile
1	Journal of Pediatric Surgery	454	9,160	2.3	Q1
2	Pediatric Surgery International	322	5,132	1.8	Q2
3	European Journal of Pediatric Surgery	99	1,485	1.8	Q2
4	Journal of Pediatric Gastroenterology and Nutrition	79	1,797	3.1	Q1
5	Journal of Pediatric Surgery Case Reports	69	130	0.2	Q4
6	Seminars in Pediatric Surgery	59	2,241	2.5	Q1
7	Neurogastroenterology and Motility	47	1,509	2.9	Q2
8	Journal of Laparoendoscopic & Advanced Surgical Techniques	44	458	1.1	Q4
9	Frontiers in Pediatrics	40	230	2.2	Q2
10	Journal of Surgical Research	33	304	1.6	Q3

Among the remaining top 10 journals, Seminars in Pediatric Surgery stands out with the highest citation count (2,241) despite ranking sixth by volume (59 articles), reflecting the high impact of its invited reviews. Notably, journals outside the core pediatric surgery discipline, including Neurogastroenterology and Motility, Journal of Laparoendoscopic and Advanced Surgical Techniques, Frontiers in Pediatrics, and Journal of Surgical Research, have also entered the top 10, suggesting growing interdisciplinary interest from gastroenterology, minimally invasive surgery, and general pediatrics communities.

### 3.5 Analysis of author influence and collaboration

Table 4 lists the top 10 authors by publication volume, all of whom published at least 20 papers. Levitt, Marc A. ranked first with 66 publications, an H-index of 46, and 1,510 total citations, followed by Puri, Prem with 57 publications, an H-index of 52, and 1,348 total citations. We constructed a collaboration network based on 46 authors with at least 15 publications. Figure 5 illustrates researchers' collaborative relationships, where node size represents publication volume, node colour indicates the collaborative cluster to which the author belongs, and line thickness reflects the frequency of co-authorship. This reveals that leading authors have formed 10 primary collaborative clusters.

**Table 4:** Top 10 authors by number of publications on the clinical treatment of Hirschsprung disease, 2005–2025. “Country” indicates the country of the author's primary institutional affiliation; “Article” the number of included publications; “H-index” the author's Hirsch index as reported by WoS; “Total citation” the total citations received; and “Total link strength” the cumulative co-authorship link strength with other authors (VOSviewer 1.6.20). Ranked by number of publications.

Rank	Author	Country	Article	H-index	Total citation	Total link strength
1	Levitt, Marc A.	United States	66	46	1,510	103
2	Puri, Prem	Ireland	57	52	1,348	66
3	Wood, Richard J.	United States	48	23	623	86
4	Goldstein, Allan M.	United States	40	38	1,418	40
5	Yamataka, Atsuyuki	Japan	37	32	272	62
6	Langer, Jacob C.	Canada	35	55	1,566	40
7	Rentea, Rebecca M.	United States	34	20	345	51
8	Feng, Jiexiong	China	32	24	394	20
9	Bischoff, Andrea	United States	30	24	504	58
10	Pakarinen, Mikko P.	Finland	29	47	1,423	29

**Figure 5:**
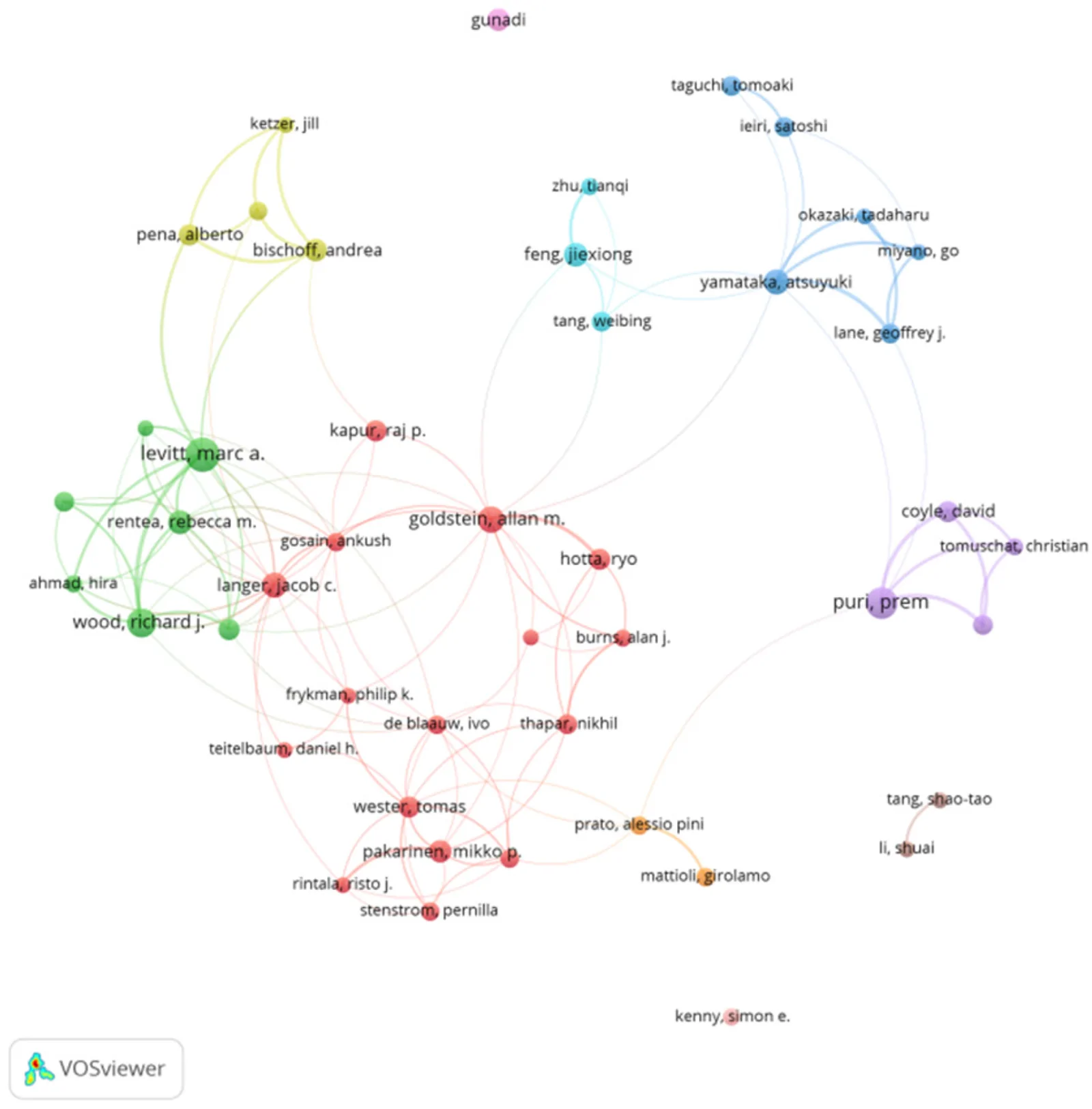
Author collaboration network for research on the clinical treatment of Hirschsprung disease, 2005–2025. The network was generated with VOSviewer 1.6.20 and is based on the 46 authors with at least 15 publications. Node size represents publication volume, node colour indicates the collaborative cluster to which the author belongs (VOSviewer clustering), line thickness reflects the frequency of co-authorship, and the resulting clusters comprise 10 primary collaborative groups.

### 3.6 Reference bursts detection

A citation burst denotes a sharp increase in citation frequency following a paper's publication, reflecting heightened scholarly interest in the corresponding topic. Figure 6 presents the top 20 references exhibiting the strongest citation bursts, in which the cyan line marks the overall citation timeline from 2005 to 2025, and the red segments denote the duration of each burst.

**Figure 6:**
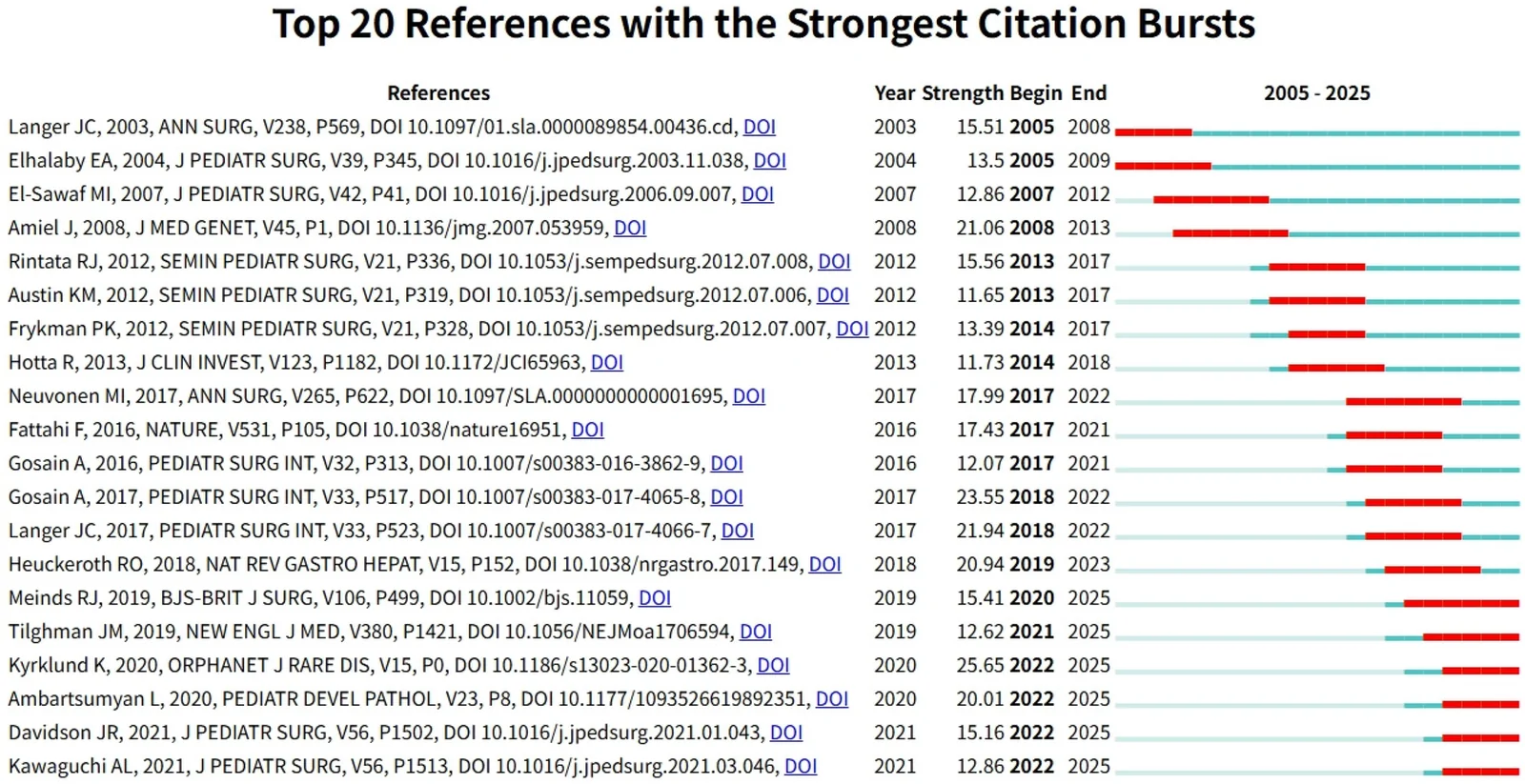
Top 20 references with the strongest citation bursts in research on the clinical treatment of Hirschsprung disease, 2005–2025. Generated with CiteSpace 6.4.R1 using 1-year slices and default parameters. The cyan line marks the full observation period (2005–2025); the red segments indicate the interval during which each reference was in burst, with segment length corresponding to burst duration. Values on the right denote burst strength; red segments reaching the right edge denote references still in burst in 2025.

The reference with the highest burst strength (25.65) was “ERNICA guidelines for the management of rectosigmoid Hirschsprung disease” (Kyrklund K, 2020), published in Orphanet Journal of Rare Diseases, with a burst period of 2022–2025. The second strongest burst (strength = 23.55) was attributed to “Guidelines for the diagnosis and management of Hirschsprung-associated enterocolitis” (Gosain A, 2017), published in Pediatric Surgery International, spanning 2018–2022.

Among the top 20 references, 6 papers continue to be in their active burst period (all extending through 2025): Meinds RJ (2019, BJS-British Journal of Surgery, 2020–2025), Tilghman JM (2019, New England Journal of Medicine, 2021–2025), Kyrklund K (2020, Orphanet Journal of Rare Diseases, 2022–2025), Ambartsoumian L (2020, Pediatric and Developmental Pathology, 2022–2025), Davidson JR (2021, Journal of Pediatric Surgery, 2022–2025), and Kawaguchi AL (2021, Journal of Pediatric Surgery, 2022–2025). The high proportion of recent publications (2019–2021) among these ongoing bursts indicates sustained and growing research interest in the field. Collectively, the burst references cluster around four major research themes: (1) the genetic basis and pathogenesis of Hirschsprung disease; (2) the prevention and management of Hirschsprung-associated enterocolitis; (3) the evaluation of long-term functional outcomes and quality of life following various surgical techniques; and (4) the emerging potential of stem cell-based therapies and regenerative medicine approaches, although currently confined to experimental/preclinical investigation.

### 3.7 Keyword analysis and analysis of keywords bursts

Keyword burst detection identifies keywords that experience a sharp increase in usage frequency over a short period, signaling emerging trends or research frontiers. Figure 7 presents the top 20 keywords with the strongest citation bursts, in which the cyan line marks the timeline from 2005 to 2025, and the red segments indicate the duration of each burst, with the start and end years corresponding to the burst period and the length of the red segments reflecting the burst duration.

**Figure 7:**
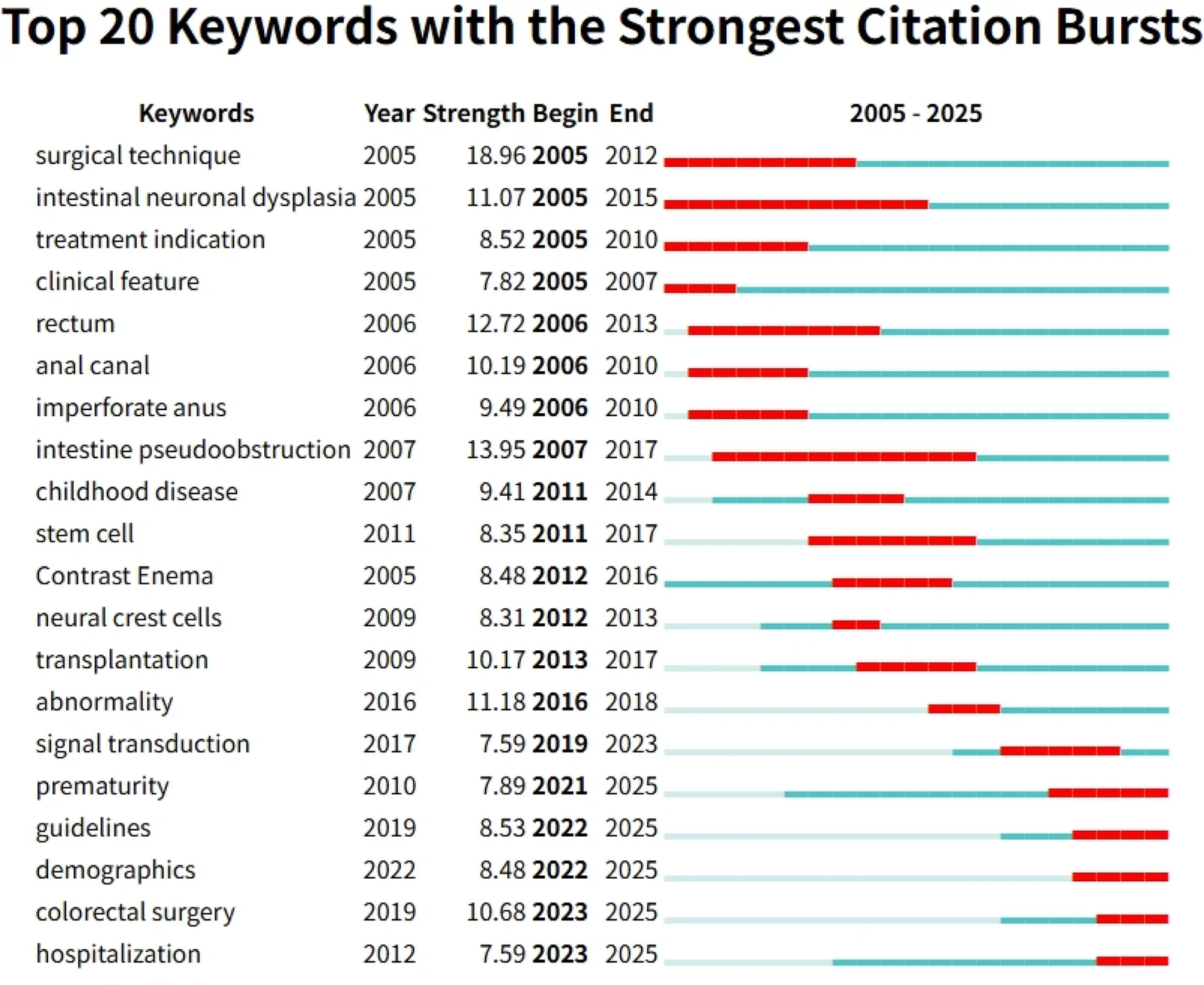
Top 20 keywords with the strongest citation bursts in research on the clinical treatment of Hirschsprung disease, 2005–2025. Generated with CiteSpace 6.4.R1 using 1-year slices and default parameters. The cyan line marks the full observation period (2005–2025); the red segments indicate the interval during which each keyword was in burst, with segment length corresponding to burst duration. Values on the right denote burst strength; red segments reaching the right edge denote keywords whose bursts remain active through 2025.

The keyword “surgical technique” attracted the highest attention, with a burst strength of 18.96 and a burst period of 2005–2012. This was followed by “intestine pseudoobstruction” (strength = 13.95, 2007–2017) and “rectum” (strength = 12.72, 2006–2013), reflecting the early focus on surgical and anatomical aspects of the disease. Notably, “intestinal neuronal dysplasia” exhibited one of the longest-lasting bursts, spanning 2005–2015 (10 years) with a strength of 11.07, indicating sustained scholarly interest in this related condition. Other keywords with relatively high burst strength include “abnormality” (strength = 11.18, 2016–2018), “colorectal surgery” (strength = 10.68, 2023–2025), “anal canal” (strength = 10.19, 2006–2010), and “transplantation” (strength = 10.17, 2013–2017).

The temporal distribution of keyword bursts reveals a clear evolution of research priorities. Early bursts (2005–2013) were dominated by anatomy- and surgery-related terms (e.g., “surgical technique”, “rectum”, “anal canal”, “imperforate anus”), reflecting the field's initial emphasis on operative approaches. The mid-period (2011–2018) saw growing attention to pathophysiology and experimental therapies, exemplified by “stem cell” (2011–2017), “transplantation” (2013–2017), and “abnormality” (2016–2018). In recent years, keywords such as “prematurity” (strength = 7.89, 2021–2025), “guidelines” (strength = 8.53, 2022–2025), “demographics” (strength = 8.48, 2022–2025), “colorectal surgery” (strength = 10.68, 2023–2025), and “hospitalization” (strength = 7.59, 2023–2025) remain active. Among the top 20 keywords, 5 are still in their burst period (extending through 2025), suggesting that contemporary research is increasingly oriented toward standardized clinical guidelines, epidemiological features, and healthcare utilization.

## 4 Discussion

### 4.1 Research landscape and knowledge base

This study employed bibliometric methods to provide an updated and more comprehensive analysis of the research landscape and developmental trajectory of Hirschsprung disease clinical treatment from 2005 to 2025, offering a structured view of the field's knowledge base, collaboration patterns, and emerging trends. A total of 3,816 publications were included in the analysis, comprising 3,201 original articles and 615 reviews. Annual publication output increased steadily from 103 in 2005 to 313 in 2025, and the annual volume has consistently exceeded the 200-mark since 2019, indicating sustained high activity in HSCR clinical treatment research. This growth trajectory is closely associated with continuous innovations in surgical techniques, refinement of complication management strategies, and the emergence of novel directions in regenerative medicine.

From a geographical perspective, a total of 106 countries/regions have contributed to research in this field, with the United States (*n* = 1,038, 27.2%), China (*n* = 495, 13.0%), and the United Kingdom (*n* = 314, 8.2%) ranking as the top three contributors, collectively accounting for nearly half of the total publications (48.4%), underscoring their leading roles in this domain.

As one of the birthplaces of pediatric surgery, the United States has maintained a prominent advantage in HSCR research by virtue of its long-standing academic tradition, well-established clinical research infrastructure, and robust funding support. China has risen to become the second-largest contributor, reflecting the sustained growth of domestic pediatric surgery and increased research investment. This rapid rise has been further driven by the concentration of a large population of children with HSCR in tertiary pediatric surgical centers and the accumulation of high-volume single-center cohorts (e.g., Huazhong University of Science and Technology, 63 publications); the progressive internationalization of Chinese pediatric surgery has translated these domestic case volumes into internationally visible output—a pattern of late entry but rapid growth that is reshaping a landscape long dominated by Europe and North America.

The United Kingdom has emerged as the third-largest contributor, distinguished by its high citation impact (9,508 total citations) and strong international collaboration. Notably, seven of the top ten high-output countries are located in Europe and the Americas, suggesting that research capacity in this field remains concentrated in developed regions, which may be closely related to the distribution of healthcare resources, the establishment of rare disease registries, and the maturity of multicenter collaborative networks.

At the institutional level, approximately 2,205 institutions worldwide have participated in HSCR clinical treatment research. Nationwide Children's Hospital (86 publications) and Huazhong University of Science and Technology (63 publications) ranked first and second, respectively. The distribution of high-output institutions generally aligns with national publication rankings; however, the rapid rise of Chinese institutions suggests that emerging economies are progressively reshaping the traditional research landscape.

Collaboration network analysis revealed that high-output authors have formed ten primary collaborative clusters, with core scholars such as Prem Puri and Marc A. Levitt not only leading in publication volume but also demonstrating central academic influence in the field, as reflected by their high H-indices and total citation counts. Nevertheless, the current international collaboration network still exhibits notable regional characteristics, and large-scale cross-continental collaboration warrants further strengthening.

In journal analysis, the Journal of Pediatric Surgery ranked first in publication volume with 454 articles, while Seminars in Pediatric Surgery, despite its lower volume, achieved the highest number of citations per article (2,241/59 ≈ 38.0) among the top 10, reflecting the high impact of its invited reviews in shaping clinical practice.

### 4.2 Evolution of research themes and emerging frontiers

Reference burst analysis revealed the dynamic evolution of the knowledge base in this field. The publication with the highest burst strength was the ERNICA guidelines for the management of rectosigmoid Hirschsprung disease (12) (strength = 25.65, burst period = 2022–2025), followed by the guidelines for the diagnosis and management of Hirschsprung-associated enterocolitis (13) (strength = 23.55, burst period = 2018–2022), the genetic study by Amiel et al. (14) (strength = 21.06, 2008–2013), the guidelines for the management of postoperative obstructive symptoms by Langer et al. (15) (strength = 21.94, 2018–2022), and the comprehensive review by Heuckeroth (16) (strength = 20.94, 2019–2023).

The prominence of guideline documents and high-impact reviews among the top burst references indicates that the development of standardized diagnostic and therapeutic frameworks has become a core concern of the academic community in recent years. Multiple high-burst references also focused on stem cell-based regenerative therapy and enteric neural regeneration, including Fattahi et al. (11) (strength = 17.43, 2017–2021) and Hotta et al. (17) (strength = 11.73, 2014–2018), suggesting that regenerative medicine strategies are attracting growing research attention, although they remain at an early preclinical stage and clinical translation has not yet been demonstrated.

Notably, six references remain under active burst through 2025, including the Meinds et al. (18) study on bowel function outcomes (strength = 15.41, 2020–2025), the Tilghman et al. (19) molecular genetic anatomy and risk-profile study (strength = 12.62, 2021–2025), the Davidson et al. (20) study on long-term surgical and patient-reported outcomes (strength = 15.16, 2022–2025), and the Kawaguchi et al. (21) systematic review on long-segment HSCR (strength = 12.86, 2022–2025), indicating that long-term functional outcome evaluation, genetic research, and standardized management of complex refractory subtypes remain persistent research priorities.

Keyword burst analysis further corroborated and refined the above phased characteristics. Over the past two decades, research hotspots in HSCR clinical treatment have exhibited a clear three-stage evolutionary trajectory.

In the first stage (approximately 2005–2012), the dominant burst keyword was “surgical technique” (strength = 18.96, 2005–2012), the highest among all keyword bursts, reflecting the academic community's intense focus on optimizing operative approaches. Concurrently, sustained bursts of “intestinal neuronal dysplasia” (strength = 11.07, 2005–2015) and “intestine pseudoobstruction” (strength = 13.95, 2007–2017) indicated that pathological mechanism research, including differential diagnosis, attracted considerable attention. Anatomical and procedural refinements were also active, with bursts of “rectum” (strength = 12.72, 2006–2013), “anal canal” (strength = 10.19, 2006–2010), and “imperforate anus” (strength = 9.49, 2006–2010) reflecting accumulating early clinical experience during the initial phase of minimally invasive technique adoption (22, 23).

In the second stage (approximately 2011–2018), research focus broadened toward diagnostic techniques, regenerative medicine, and long-term assessment, evidenced by bursts of “Contrast Enema” (strength = 8.48, 2012–2016), “transplantation” (strength = 10.17, 2013–2017), “stem cell” (strength = 8.35, 2011–2017), and “abnormality” (strength = 11.18, 2016–2018). The emergence of stem cell and transplantation-related keywords marked the early transition of regenerative medicine strategies from basic research toward clinical application, albeit still at a preclinical stage (10, 11, 17).

In the third stage (2019–present), the active bursts of keywords including “guidelines” (strength = 8.53, 2022–2025), “colorectal surgery” (strength = 10.68, 2023–2025), “prematurity” (strength = 7.89, 2021–2025), “demographics” (strength = 8.48, 2022–2025), “hospitalization” (strength = 7.59, 2023–2025) indicate that current research is progressively shifting toward the development and dissemination of standardized diagnostic and therapeutic consensus, refinement of colorectal surgical techniques, and management of HSCR in premature and special populations (24). Some of these burst keywords (e.g., “abnormality,” “demographics,” “hospitalization,” “intestine pseudoobstruction”) are relatively generic or denote adjacent clinical entities; their prominence therefore reflects broader research activity rather than HSCR-specific hotspots and should be interpreted with caution.

Taken together, the field has undergone a progressive evolution from “surgical technique optimization and pathological exploration” to “regenerative medicine and long-term assessment”, and subsequently to “standardized management and individualized treatment of children with complex disease”. The arrival of this final stage is best understood as a response to a specific turning point rather than a gradual drift. It was precipitated by the coordinated publication of formal consensus frameworks—most prominently the ERNICA guidelines for rectosigmoid HSCR (2020) and the HAEC management guidelines (2017), which correspond to the two strongest reference bursts in our analysis (strengths 25.65 and 23.55, respectively)—followed by international and national consensus documents on minimally invasive pull-through. These guidelines addressed a recognized gap: two decades of surgical innovation had accumulated without harmonized standards, leaving wide institutional variability in technique, perioperative care, and follow-up. The European Reference Networks for rare diseases supplied the organizational vehicle for this standardization. The “guideline era” is therefore a collective response to variability—and to the need to measure long-term outcomes against shared benchmarks—rather than a mere shift in keyword vocabulary.

### 4.3 Critical appraisal and recommendations

An important caveat follows from our methods. Citation and keyword bursts measure research attention—the intensity and timing of scholarly activity—not the quality or maturity of the underlying evidence. We therefore distinguish “research heat” from “evidence maturity” when appraising the hotspots identified above.

The current hotspots differ sharply in their evidence base. HAEC prevention is the field's most clinically urgent problem, yet its evidence remains dominated by single-center retrospective cohorts, case series, and expert consensus; a recent meta-analysis of risk factors (10,012 cases) consolidates prognostic knowledge, but preventive interventions (irrigation protocols, probiotics, botulinum toxin) still lack robust randomized comparisons, and HAEC itself lacks a universally accepted definition and severity grading. Long-term functional follow-up and quality-of-life research, though among the most cited active themes, similarly consists largely of cross-sectional and single-center longitudinal studies with heterogeneous outcome measures, limiting meta-analytic synthesis.

Guideline implementation, the signature theme of the current era, rests on recommendations that—as the ERNICA document itself acknowledges—are predominantly expert-opinion based (GRADE low/very-low; Canadian Task Force level III), reflecting the scarcity of high-quality evidence in a rare disease. Stem-cell and regenerative approaches, despite generating the most striking conceptual excitement, remain strictly at the preclinical/*ex vivo* stage; even the most advanced demonstration to date (transplantation of human pluripotent stem-cell-derived enteric nervous system progenitors into tissue obtained from patients with HSCR) improves contractility in explanted tissue but has not been tested *in vivo*, and clinical translation has not yet been demonstrated. Precision surgery for long-segment and total colonic aganglionosis faces the dual challenge of the small number of affected children and heterogeneous anatomy, which has thus far precluded definitive comparative evidence.

To translate these findings into action, we offer the following concrete, evidence-based recommendations rather than generic calls for “more collaboration.” First, establish multicenter prospective registries modeled on the ERNICA/APSA-based networks, with standardized data dictionaries and outcome measures, to convert the field's concentrated single-center experience into comparable, pooled evidence—the single highest-leverage intervention for a rare disease. Second, prioritize the standardization of HAEC definition and severity grading (e.g., a Delphi-derived core outcome set) and evaluate preventive strategies in pragmatic prospective designs, since prevention—not rescue—is where morbidity is most tractably reduced. Third, invest in protocolized longitudinal functional follow-up extending into adulthood, including formal transition-of-care pathways, to quantify and mitigate soiling, constipation, fertility, and quality-of-life sequelae that persist well beyond the surgical event. Fourth, design comparative-effectiveness studies (and, where feasible, pragmatic randomized trials) of competing surgical techniques and perioperative protocols, leveraging the registry infrastructure above. Fifth, channel regenerative medicine through a defined translational pathway—from the current *ex vivo* demonstrations toward standardized *in vivo* preclinical models and, ultimately, carefully staged early-phase trials—so that its promise is tested against explicit safety and efficacy milestones rather than announced prematurely. Finally, cross-continental collaboration between established and emerging centers should be organized around these shared priorities rather than pursued for its own sake.

### 4.4 Limitations

Although bibliometrics provides objective data support for mapping the broader landscape of the field, several limitations of this study should be acknowledged. First, although this study combined two major databases (Web of Science Core Collection and Scopus), other databases such as EMBASE and Google Scholar were not included, which may have led to the omission of certain relevant publications. Second, the language restriction to English may have introduced selection bias by excluding high-quality non-English studies. Third, bibliometric analysis inherently carries a certain degree of lag, as burst keywords reflect research directions that have already garnered attention, and may not be sufficiently sensitive to nascent frontier topics. Fourth, although 1,899 duplicate records were removed through DOI and title-based deduplication, some residual duplicates or near-duplicates may persist due to inconsistencies in bibliographic metadata across databases. Future research could consider integrating multiple data sources and combining expert consultation with clinical registry analyses to achieve earlier identification and more comprehensive capture of emerging frontiers.

In summary, this study provides a systematic bibliometric mapping of the knowledge landscape and evolutionary trajectory of research hotspots in the field of Hirschsprung disease clinical treatment. The field has currently established a core research force comprising the United States, China, and the United Kingdom, and has undergone a three-stage evolution from “surgical technique optimization” through “pathophysiology and experimental therapies” to “standardized management and individualized treatment”. The development of standardized diagnostic and therapeutic frameworks, along with comprehensive HAEC management, constitutes the current research priorities, while stem cell-based regenerative therapy, precision surgical strategies for long-segment Hirschsprung disease, and the establishment of long-term postoperative follow-up systems represent important frontiers at an early experimental stage warranting focused attention in the future.

Pediatric surgery researchers should prioritize multicenter prospective registries, standardized HAEC definition and prevention, and protocolized long-term follow-up as concrete levers for advancing the field. Owing to differences in search syntax between databases, the WOSCC query included additional synonyms (“congenital megacolon,” “congenital aganglionosis,” “aganglionosis”) that were omitted from the Scopus query, which may have introduced minor selection bias; however, the core search concepts were consistent across both databases and this is unlikely to materially affect the main conclusions.

## 5 Conclusions

This study systematically reviewed the research progress in the field of Hirschsprung disease clinical treatment from 2005 to 2025 using bibliometric methods. The findings revealed a sustained increase in publication output over the past two decades, with the United States, China, and the United Kingdom identified as the core research forces, and the Journal of Pediatric Surgery as the most influential journal in this field. The research themes have undergone a three-stage evolution, progressing from “surgical technique optimization” through “pathophysiology and experimental therapies” to “standardized management and individualized treatment”. Current research hotspots are concentrated on comprehensive prevention and management of Hirschsprung-associated enterocolitis (HAEC), long-term functional follow-up, and the development of standardized diagnostic and therapeutic guidelines.

Stem cell-based regenerative therapy and precision surgical strategies for long-segment/total colonic aganglionosis represent important future frontier directions warranting further investigation. The above findings may serve as a reference for pediatric surgeons and researchers in grasping the dynamics of the field and optimizing research prioritization. In the future, multicenter prospective registries, standardized HAEC definition and prevention, and protocolized long-term follow-up should be prioritized to facilitate the generation of high-quality clinical evidence.

## Data Availability

The data analyzed in this study were obtained from the Web of Science Core Collection and Scopus databases. Restrictions apply to the availability of these data because they were used under license for the current study and are therefore not publicly available. The data are available from the corresponding author upon reasonable request. Detailed search strategies are provided in the Methodology.
